# Insights Into circRNA‐Mediated Lipid Metabolism in Cancer Progression

**DOI:** 10.1111/jcmm.71052

**Published:** 2026-02-07

**Authors:** Zhiwei Miao, Jingjing Cao, Xiaoyu Wang, Chunyu Zhang, Tongguo Shi

**Affiliations:** ^1^ Department of Gastroenterology Zhangjiagang TCM Hospital Affiliated to Nanjing University of Chinese Medicine Zhangjiagang China; ^2^ Jiangsu Institute of Clinical Immunology, The First Affiliated Hospital of Soochow University Suzhou China

**Keywords:** cancer, cholesterol, circRNA, fatty acids, lipid metabolism

## Abstract

Lipid metabolism reprogramming is one of the most prominent characteristics of cancer, but it is still unclear which regulatory pathways underlie this process in cancer cells. Circular RNAs (circRNAs) represent a novel category of non‐coding RNAs with multifaceted regulatory functions. While the biological roles of circRNAs in cancer have been elucidated, there remains a dearth of knowledge regarding their involvement and regulatory mechanisms in lipid metabolism within the context of cancer. This article provides an overview of the regulatory roles and specific mechanisms of circRNAs in cancer lipid metabolism reprogramming. By elucidating these mechanisms, it enhances our comprehension of the metabolic rewiring driving tumour development and uncovers new avenues for targeted therapy.

## 1 Introduction

Lipids, commonly referred to as fats, encompass a diverse array of molecules, including fatty acids, cholesterol, phospholipids, triglycerides and sphingolipids [1]. It is well established that a lipid is a fundamental building block of a cell and a critical component of its membrane. In addition to their structural roles, the function of lipids is to transmit intracellular signals and act as energy carriers under nutrient‐limited conditions [2, 3]. Lipid metabolism, which involves the uptake, synthesis and hydrolysis of lipids, is critical to maintain cellular homeostasis, and its dysregulation is implicated in the pathogenesis and progression of various diseases, including diabetes, Alzheimer's disease, obesity and cancers [4, 5, 6]. Reprogramming of lipid metabolism has long been recognised as a hallmark of malignant tumours [4, 7]. Emerging evidence indicates that cancer cells can alter lipid metabolism through multiple signalling pathways, thereby promoting rapid growth and proliferation of tumour cells [7]. Nonetheless, a deeper understanding of the lipid metabolism reprogramming mechanisms in cancer cells is still lacking.

Non‐coding RNAs (ncRNAs), including microRNAs (miRNAs), long non‐coding RNAs (lncRNAs) and circular RNAs (circRNAs), have been implicated in the modulation of various biological functions, including lipid metabolism [8, 9, 10]. While miRNAs and lncRNAs have been associated with the tumour cell reprogramming of lipid metabolism [11, 12], it is unclear how circRNAs are involved in cancer lipid metabolism and how they are regulated. Based on recent research, this review offers new insights into the mechanisms by which differentially expressed circRNAs contribute to lipid metabolism reprogramming in multiple cancers.

## 2 Lipid Metabolism Reprogramming in Cancer

Altered metabolism is a key hallmark of cancer, with metabolic reprogramming providing tumour cells with an increased supply of nutrients. Unlike normal cells that primarily rely on the uptake of exogenous fatty acids (FA), cancer cells enhance de novo fatty acid synthesis. A fatty acid comprises a carboxyl terminal group and an elongated hydrocarbon chain. It is essential for fatty acid metabolism to have processes of fatty acid synthesis, transportation and oxidation (FAO) [13]. The synthesis of fatty acid occurs in the cellular cytosol and involves multiple sequential enzymatic reactions. Acetyl‐CoA, produced via the tricarboxylic acid (TCA) cycle, the pentose phosphate pathway (PPP) and glycolysis, is carboxylated into malonyl‐CoA by acetyl‐CoA carboxylases (ACCs). Subsequently, one acetyl‐CoA molecule and seven malonyl‐CoA molecules condense to form saturated palmitate (FA16:0) under the catalytic action of fatty acid synthase (FASN). Through elongation and desaturation processes, palmitate is further converted into various saturated and unsaturated long‐chain fatty acids (LCFAs) [14]. The transportation of fatty acids involves their direct uptake by cells from the surrounding microenvironment. This process is mediated by fatty acid translocase (FAT, also known as CD36), plasma membrane fatty acid‐binding proteins (FABP) and the fatty acid transport protein family (FATPs, also known as SLC27) [15, 16]. Long‐chain fatty acids undergo fatty acid oxidation within mitochondria to produce acetyl‐CoA, nicotinamide adenine dinucleotide (NADH) and flavin adenine dinucleotide (FADH2). As acyl‐CoA and carnitine are converted into acyl‐carnitine by carnitine palmitoyltransferase I (CPT1), its rate‐limiting function is to facilitate this process, which is then transported from the cytosol to the mitochondrial matrix. The FADH2 and NADH produced are subsequently utilised in the electron transport chain for ATP synthesis [17, 18].

Another important biosynthetic process related to cancer is the mevalonate pathway, which mediates cholesterol synthesis. The cholesterol biosynthetic pathway involves approximately 30 enzymatic reactions that convert acetyl‐CoA into cholesterol. Two critical rate‐limiting enzymes, 3‐hydroxy‐3‐methylglutaryl‐CoA (HMG‐CoA) reductase (HMGCR) and squalene epoxidase (SQLE), are located in the endoplasmic reticulum. HMGCR is responsible for converting HMG‐CoA to mevalonate (MVA), while squalene is oxidised to 2,3‐epoxysqualene by SQLE [19, 20]. It is well known that receptor‐mediated uptake of exogenous cholesterol accounts for most cellular cholesterol, specifically high‐density lipoprotein cholesterol (HDL‐C) through selective uptake and low‐density lipoprotein cholesterol (LDL‐C) via endocytosis [4, 21]. The LDL receptor‐mediated endocytosis pathway is essential for cholesterol uptake. After binding to the cell membrane, LDL particles and LDL receptors are internalised and transported to lysosomes, where free cholesterol is released from them after they are hydrolyzed by acid lipase. The selective uptake pathway for cholesterol primarily involves HDL‐derived cholesterol and operates independently of endocytosis. HDL and cholesterol esters are internalised into the cell membrane through the hydrophobic channel of the HDL receptor, scavenger receptor class B type 1 (SR‐B1) and transported to organelles, such as the endoplasmic reticulum [22, 23]. Cholesterol homeostasis in cells is predominantly regulated by two transcription factors: sterol regulatory element‐binding protein 2 (SREBP2) and liver X receptors (LXRs) [4].

**FIGURE 1:**
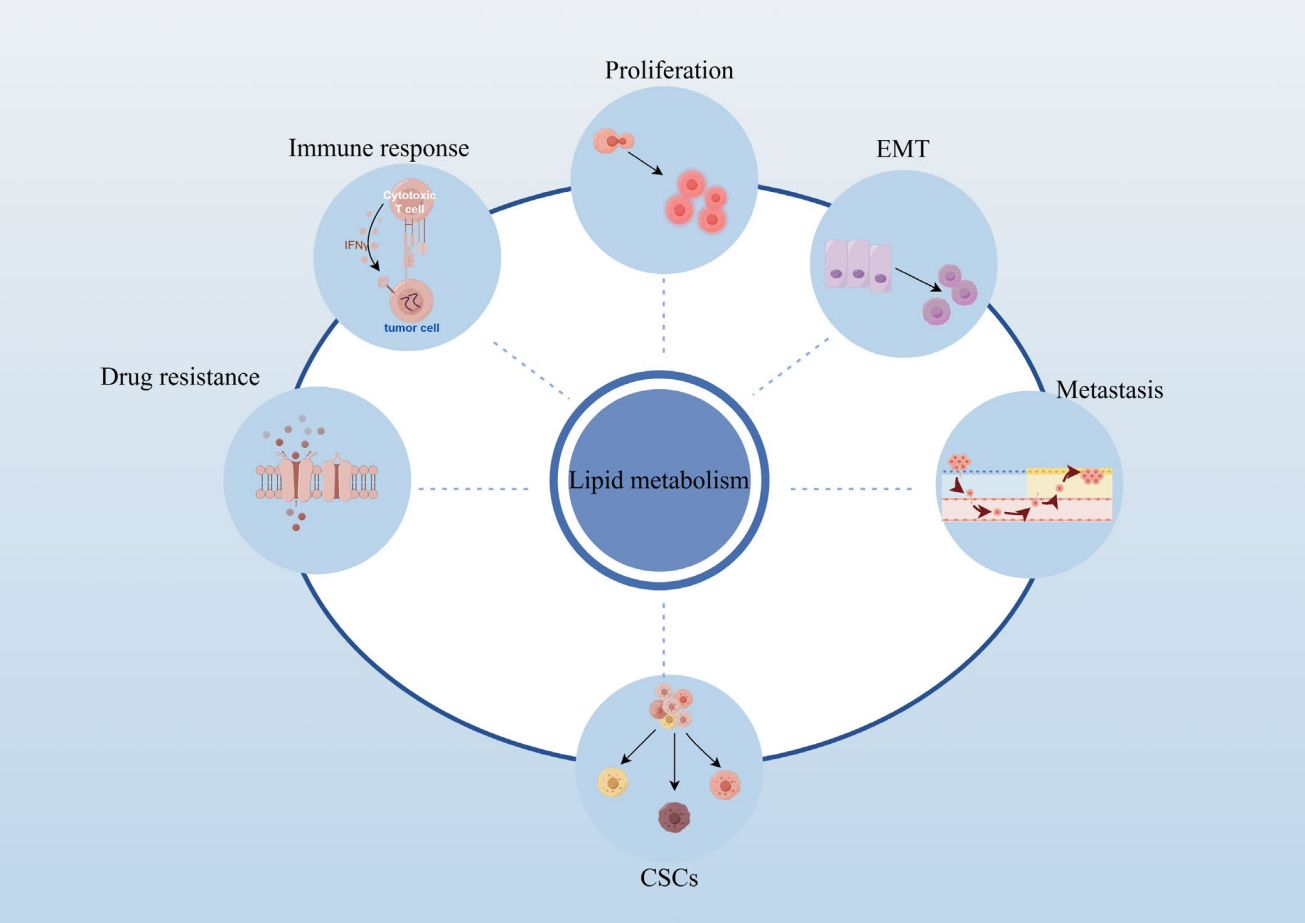
The biological functions of lipid metabolism in cancer. Lipid metabolism plays crucial roles in modulating tumour cell proliferation, EMT, metastasis, CSCs, drug resistance, and immune response. The graphic was created using Figdraw (www.figdraw.com).

In summary, lipid metabolic reprogramming has garnered significant research attention in cancer biology due to its profound effects on key cancer behaviours. It plays a critical role in promoting tumour cell proliferation, inducing epithelial‐mesenchymal transition (EMT), facilitating metastasis, contributing to drug resistance and modulating immune responses (Figure 1), making it a vital area of study for understanding and combating cancer.

## 3 Effect of Lipid Metabolism on Cancer Cell Proliferation

Lipid metabolic reprogramming fuels tumour proliferation by supplying essential building blocks and bioactive molecules for rapid cancer cell growth and survival [24]. In cancer cells, lipid metabolic pathways are extensively rewired to meet the heightened demands of cell division and expansion. For instance, the glycolytic enzyme PFKL governs lipolysis by promoting lipid droplet‐mitochondria tethering, thereby enhancing β‐oxidation and tumour cell proliferation [25]. Additionally, RNA‐binding motif protein 45 (RBM45) promotes lipid utilisation in hepatocellular carcinoma (HCC) cells and drives HCC proliferation via the Rictor and ACSL1/ACSL4 axis [26].

### 3.1 Effect of Lipid Metabolism on EMT and Metastasis of Cancer Cells

Beyond supporting proliferation, lipid metabolism also plays a crucial role in EMT and cancer metastasis [27]. During EMT, tumour cells dramatically alter their membrane composition and fluidity to enhance migration and invasion [28]. Changes in lipid metabolism, like boosted de novo lipid synthesis and shifts in lipid composition, preserve membrane fluidity and integrity. This supports the cell shape and motility changes needed for EMT and metastasis [29]. For example, Zhou et al. showed that cordycepin blocks cholangiocarcinoma metastasis and EMT by reprogramming lipid metabolism via the ERO1A/mTOR/SREBP1 axis [30]. Also, TGFβ2‐induced lipid droplet formation drives acidosis‐related EMT and cancer cell spread [31]. Furthermore, cancer‐derived exosomal HSPC111 promotes colorectal cancer liver metastasis by altering lipid metabolism in cancer‐associated fibroblasts [32].

### 3.2 Effect of Lipid Metabolism on Cancer Stem Cells

Lipid metabolic reprogramming is also crucial for cancer stem cells (CSCs), which are responsible for tumour initiation and propagation [33]. CSCs display enhanced de novo lipogenesis and FAO [33]. For instance, NANOG, a key CSC regulator, stimulates FAO to meet tumour‐initiating cells' energy demands and shifts metabolism from OXPHOS to FAO, thereby strengthening CSC self‐renewal and tumorigenic potential [34]. The ncRNA lncROPM maintains breast CSCs by directly binding to PLA2G16 mRNA's 3′‐UTR, stabilising it and boosting PLA2G16 expression. This elevation enhances phospholipid metabolism and liberates fatty acids like arachidonic acid, subsequently activating PI3K/AKT, Wnt/β‐catenin and Hippo/YAP signalling pathways [35]. Overall, lipid metabolic reprogramming is vital for sustaining CSC properties and functions, presenting potential therapeutic targets to disrupt CSC metabolism and enhance cancer therapy.

### 3.3 Effect of Lipid Metabolism on Cancer Drug Resistance

Lipid metabolism further impacts cancer by influencing drug resistance. It achieves this by altering cell membrane composition and fluidity. For example, Shimolina et al. observed that 5‐fluorouracil‐resistant cells have lower monounsaturated fatty acid levels and increased sphingomyelin or decreased phosphatidylcholine in their membranes, which increases viscosity and contributes to drug resistance [36]. Lipid‐derived signalling molecules also activate prosurvival pathways like PI3K/AKT, enhancing cell survival under treatment stress. Specifically, 14,15‐epoxyeicosatrienoic acid (14,15‐EET) induces EMT and cisplatin resistance in breast cancer cells by upregulating integrin αvβ3 and activating FAK/PI3K/AKT signalling [37]. Additionally, cancer cells use lipid metabolism to increase energy production through fatty acid oxidation, supporting survival during drug challenge. For instance, low GPR81 in ER+ breast cancer cells drives tamoxifen resistance by inducing PPARα‐mediated fatty acid oxidation [38]. Targeting lipid metabolism may offer a strategy to overcome drug resistance and improve cancer therapy.

### 3.4 Effect of Lipid Metabolism on Cancer Immune Responses

Lipid metabolism significantly impacts tumour immune responses by modulating immune cell activity and the tumour microenvironment [39, 40]. For instance, lysophosphatidic acid (LPA) can inhibit CD8+ T‐cell function and anti‐tumour immunity by modulating metabolic efficiency through LPAR5 signalling [41]. Conversely, Miao et al. demonstrated that the tRNA m1A ‘writer’ gene Trmt61a enhances the tumour‐killing capacity of CD8+ T cells by regulating cholesterol biosynthesis [42]. Moreover, lipid metabolism affects the tumour microenvironment by influencing tumour‐associated macrophages, T cells, dendritic cells and myeloid‐derived suppressor cells, thereby contributing to an immunosuppressive environment [43]. These insights highlight the potential of targeting lipid metabolism to enhance antitumor immunity and improve cancer treatment outcomes.

Overall, lipid metabolism reprogramming is a crucial element in cancer biology, significantly influencing tumour progression through multiple mechanisms. Targeting and regulating lipid metabolism in cancer may thus be effective strategies to enhance cancer therapies. Recent studies have revealed that circRNAs play a key role in modulating lipid metabolism in cancer. Then, our focus narrows to circRNAs, exploring their roles and mechanisms in regulating lipid metabolism within cancer.

## 4 circRNAs Are Key Regulators of Lipid Metabolism

**FIGURE 2:**
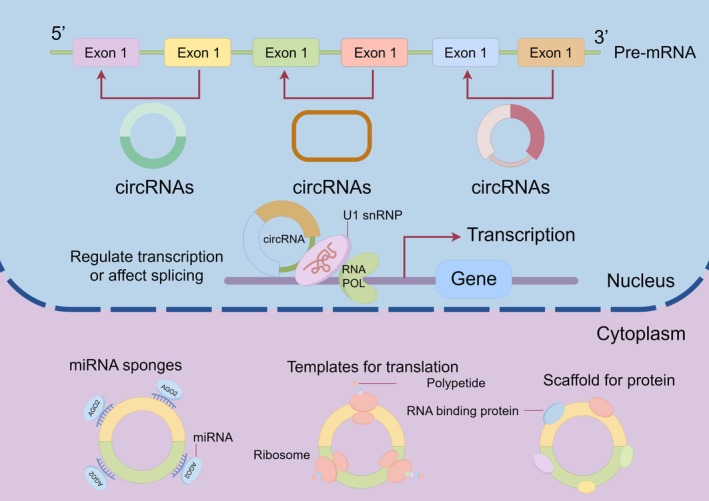
CircRNAs are formed through back‐splicing of pre‐mRNA, distinguishing them from linear mRNAs produced by canonical splicing. In the nucleus, circRNAs can regulate transcription by interacting with chromatin or transcription factors. Within the cytoplasm, circRNAs exert diverse biological effects. They can act as miRNA sponges, sequestering miRNAs and relieving their inhibitory effects on target genes. CircRNAs can also serve as templates for translation, producing functional proteins. Moreover, they can function as protein scaffolds, facilitating the assembly of protein complexes. The graphic was created using Figdraw (www.figdraw.com).

In light of the rapid advancement of next‐generation sequencing (NGS) and innovative bioinformatics techniques, circRNAs have garnered significant attention from researchers worldwide [44]. CircRNAs, which lack 5′ caps and 3′ ends, are generated from linear pre‐messenger RNAs (mRNAs) through a back‐splicing mechanism [45]. Although the precise mechanisms underlying circRNA biogenesis require further investigation, these ncRNAs can be categorised into four types: intron circRNAs, exon‐intron circRNAs, exon circRNAs and intergenic circRNAs (Figure 2) [46]. Recent studies have identified three key biological characteristics of circRNAs: (1) their covalently closed loop structures confer high stability; (2) most circRNAs exhibit high conservation across different species; and (3) they are widely present in various species. These properties render circRNAs valuable as biomarkers or therapeutic targets in clinical settings [47, 48].

Recent accumulating evidence suggests that circRNAs function as critical regulators in various cancers. For example, the intergenic circRNA circ_0007379 modulated miR‐320a biogenesis to suppress colorectal cancer (CRC) progression in a KH‐type splicing regulatory protein (KSRP)‐dependent manner [49]. Similarly, CircPDIA4 acts as a decoy by interacting with DExH‐box helicase 9 (DHX9), inhibiting its repressive effects on circRNA biogenesis, thereby enhancing the expression of multiple oncogenic circRNAs and promoting gastric cancer (GC) progression [50]. CircRNAs are implicated in regulating a variety of biological functions under both physiological and pathological conditions through mechanisms such as regulating transcription, splicing, translation and translational regulation, serving as protein reservoirs or interacting with RNA‐binding proteins (RBPs), and acting as miRNA sponges (Figure 2) [44, 51]. Despite these findings, it is largely unknown what role circRNAs play in modulating lipid metabolism reprogramming in cancer.

### 4.1 circRNAs Modulate Lipid Transportation‐Associated Genes

**FIGURE 3:**
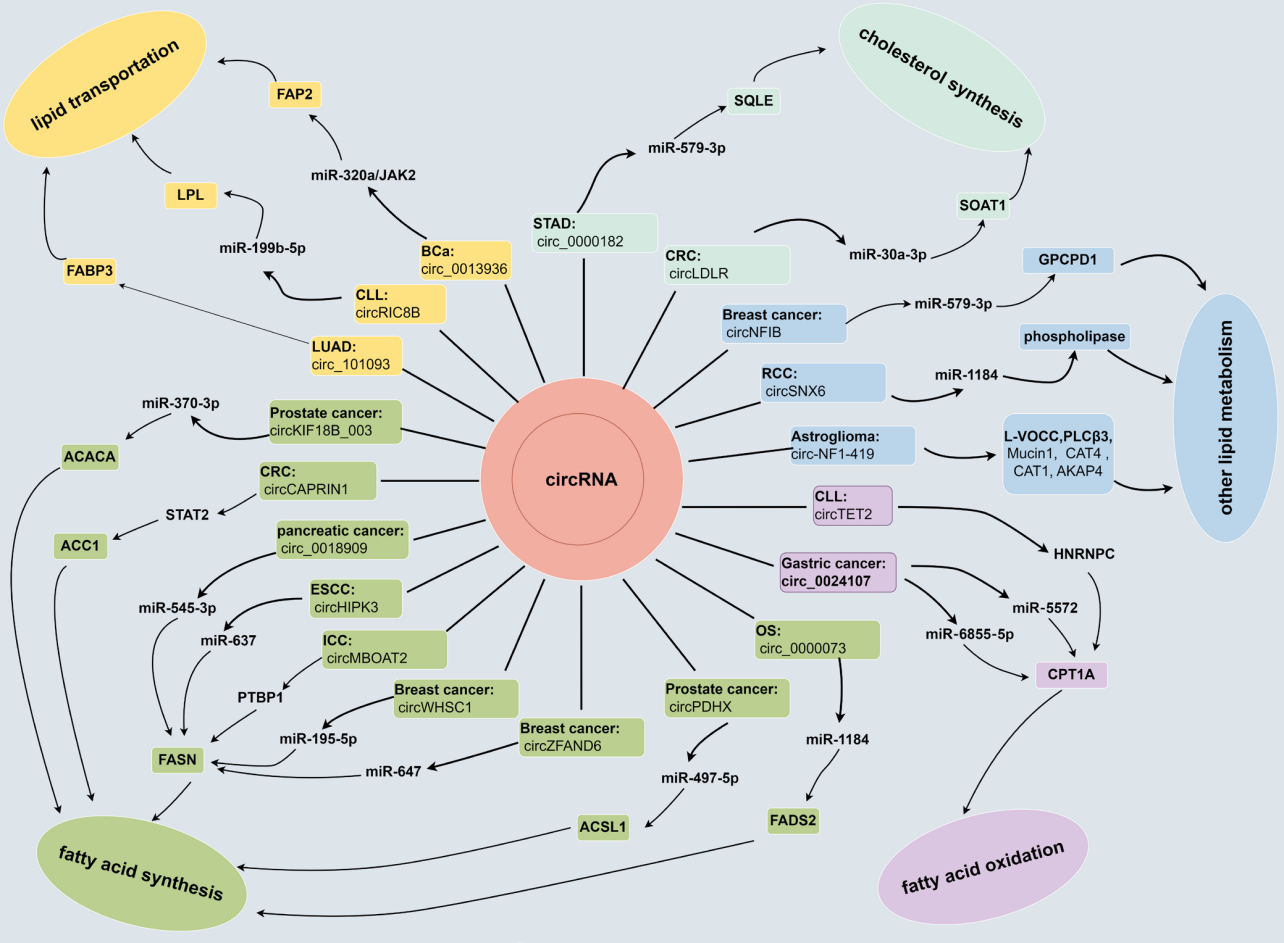
CircRNAs modulate lipid metabolism reprogramming in cancer. These circRNAs regulate the lipid transportation, fatty acid synthesis, fatty acid oxidation, cholesterol synthesis and other lipid metabolism. The graphic was created using Figdraw (www.figdraw.com).

Fatty acid and cholesterol transport mechanisms within cells are critical to maintaining lipid metabolism balance [13, 15, 16]. Particularly, proliferating tumour cells often regulate levels of proteins associated with lipid transport to enhance uptake of fatty acids and cholesterol [52, 53]. Consequently, strategies targeting lipid transport pathways could potentially inhibit cancer progression. It has been discovered that circRNAs regulate the expression of genes related to lipid transport in cancer contexts (Table 1 and Figure 3).

**TABLE 1:** Summary of circRNAs involved in lipid metabolism reprogramming in tumours.

CircRNAs	Cancer types	Expression change	Cell line	In vivo model	Functions	Possible mechanism	References
circRNA_0013936	Bladder cancer	Up	PMN‐MDSCs	/	Regulate lipid transportation	miR‐320a/JAK2/FATP2 aixs	[54]
circRIC8B	CLL	Up	MEC‐1 JVM‐3	/	Regulate lipid transportation	miR‐199b‐5p/LPL axis	[55]
circRNA_101093	LUAD	Up	H1957 A549	A549 xenograft patient‐derived xenograft	Regulate lipid transportation	Interacting with FABP3	[56]
circABCA1	ccRCC	Up	Caki‐1 786‐O	Caki‐1 xenograft 786‐O xenograft Hepatic metastasis model	Regulate cholesterol transportation	Interacting with IGF2BP3	[57]
circKIF18B_003	prostate cancer	Up	DU145 PC3	PC‐3 xenograft	Regulate fatty acid synthesis	miR‐370‐3p/ACACA axis	[58]
circCAPRIN1	CRC	Up	LoVo RKO	LoVo xenograft RKO xenograft HCT116 in vivo metastasis	Regulate fatty acid synthesis	Interacting with STAT2 and activate ACC1 transcription	[59]
circPCNXL2	PTC	Up	TPC‐1 KTC‐1	TPC‐1 xenograft	Regulate fatty acid synthesis	interacting with ACC1	[60]
circ_0018909	Pancreatic cancer	Up	SW1990 PANC‐1	SW1990 xenograft	Regulate fatty acid synthesis	miR‐545‐3p/FASN axis	[61]
circHIPK3	ESCC	Up	KYSE140 EC9706	KYSE140 xenograft	Regulate fatty acid synthesis	miR‐637/FASN axis	[62]
circMBOAT2	ICC	Up	RBE HCCC‐9810	RBE xenograft	Regulate fatty acid synthesis	Interacting with PTBP1 and facilitated FASN mRNA cytoplasmic export	[63]
circWHSC1	Breast cancer	Up	MCF7 MDA‐MB‐231	MCF7 xenograft MDA‐MB‐231 xenograft	Regulate fatty acid synthesis	miR‐195‐5p/FASN axis	[64]
circZFAND6	Breast cancer	Up	MCF7 MDA‐MB‐231	Lung metastasis model	Regulate fatty acid synthesis	miR‐647/FASN axis	[65]
circPDHX	PCa	Up	22RV1 PC3	PC‐3 xenograft	Regulate fatty acid synthesis	miR‐497‐5p/ACSL1 axis	[66]
circ_0000073	OS	Up	MG‐63 Saos‐2	MG‐63 xenograft	Regulate fatty acid synthesis	miR‐1184/FADS2 axis	[67]
circ_0000182	STAD	Up	BGC‐823 AGS	/	Regulate cholesterol synthesis	miR‐579‐3p/SQLE axis	[68]
circLDLR	CRC	Up	RKO HCT116 HT29 SW480	HCT116 xenograft HT29 xenograft	Regulate cholesterol esters	miR‐30a‐3p/SOAT1 axis	[69]
circNFIB	Breast cancer	Down	MCF7 MDA‐MB‐231	MCF7 xenograft	Regulate arachidonic acid synthesis	Regulating phospholipase	[70]
circSNX6	RCC	Up	ACHN 786‐O	786‐O xenograft	Regulate lysophosphatidic acid	miR‐1184/GPCPD1 axis	[71]
circ_0024107	Gastric cancer	/	HGC‐27 AGS	Lymph node metastasis model	Regulate fatty acid oxidation	miR‐5572/miR‐6855‐5p/CPT1A axis	[72]
circTET2	CLL	Up	MEC‐1 JVM‐3	/	Regulate fatty acid oxidation	Interacting with HNRNPC and modulated CPT1A stability	[73]
circ‐NF1‐419	Astroglioma	/	U87 U87‐NF1‐419	U87 xenograft	Regulate glycerophospholipid metabolism	Regulate the expression of metabolic lipid enzymes	[74]
circPRKAA1	HCC	Up	Huh7 PLC/PRF/5	PLC/PRF/5 xenograft	Influence regulators of lipid metabolism	Enhance the stability of mSREBP‐1	[75]
circMyc	TNBC	Up	BT‐20 MDA‐MB‐231	BT‐20 xenograft MDA‐MB‐231 xenograft	Influence regulators of lipid metabolism	Increasing SREBP1 mRNA stability and SREBP1 transcription	[76]
circINSIG1	CRC	Up	HCT8 DLD1	DLD1 orthotopic xenograft CRC PDX model	Influence regulators of cholesterol biosynthesis	Encoded protein circINSIG1‐121 and promoted degradation of INSIG1	[77]
circ_0086414	ccRCC	Down	768‐O A498	A498 xenograft	Influence regulators of lipid metabolism	Modulating the miR‐661/ERBB2/PLIN3 axis	[78]
circACC1	CRC	Up	HCT116 LO2	HCT116 xenograft	Regulate cancer‐related signalling pathways	Stabilise and enhance the enzymatic activity of the AMPK	[79]
circLARP1B	HCC	Up	PLC/PRF/5 HepG2	Diethylnitrosamine‐induced HCC mouse model	Regulate cancer‐related signalling pathways	HNRNPD–LKB1–AMPK pathway	[80]
circ_63706	MB	/	DAOY ONS76 UW228	DAOY xenograft ONS76 xenograft	Regulate cancer‐related signalling pathways	/	[81]
circARL8B	Breast cancer	Up	MCF7 MDA‐MB‐231	MCF7 xenograft	Affect lipid metabolism by regulating other factors	miR‐653‐5p/HMGA2 axis	[82]
circFAM126A	PCa	Up	PC‐3 DU145	PC‐3 xenograft	Affect lipid metabolism by regulating other factors	miR‐505‐3p/CANX	[83]

Fatty acid transport protein 2 (FATP2), belonging to the fatty acid transport protein family, functions both as a very long‐chain acyl‐coenzyme A synthetase (ACSVL) to activate long‐chain fatty acids (LCFAs) and as a transporter for LCFAs [84]. Elevated FATP2 expression has been implicated as an oncogenic factor in various cancers including bladder cancer (BCa), non‐small cell lung cancer (NSCLC), melanoma and thyroid carcinoma [85, 86, 87, 88]. According to Shi et al., exosomal circRNA_0013936 derived from BCa cells enhances immunosuppressive functions in polymorphonuclear myeloid‐derived suppressor cells (PMN‐MDSCs) by downregulating receptor‐interacting serine/threonine kinase 3 (RIPK3) through the axis involving circRNA_0013936, miR‐301b and cAMP responsive element binding protein 1 (CREB1) [54]. Simultaneously, it impairs CD8^+^ T cell functions by upregulating FATP2 via the pathways circRNA_0013936/miR‐320a/JAK2 [54].

Lipoprotein lipase (LPL) is crucial in cancer cell metabolism, increasing cellular uptake of lipoproteins and accelerating triglyceride hydrolysis into free fatty acids [89]. In chronic lymphocytic leukaemia (CLL), circRIC8B, increased in cancer tissues, enhances CLL cell proliferation and lipid accumulation by upregulating LPL in a miR‐199b‐5p‐dependent manner [55]. FABPs, a family of lipid chaperones, facilitate cellular fatty acid solubilisation, transport and metabolism [90]. Among them, FABP3 is pivotal for transporting polyunsaturated fatty acids (PUFAs) such as arachidonic acid (AA) [91]. Zhang et al. demonstrated that circRNA_101093, which is upregulated in lung adenocarcinoma (LUAD) tissues, interacts with FABP3. This interaction elevates FABP3 levels, desensitises LUAD cells to ferroptosis in vitro and in vivo, and thereby promotes the transport and subsequent taurine‐conjugation of arachidonic acid [56].

Scavenger Receptor Class B Member 1 (SCARB1) is a critical mediator of cholesterol uptake in cancer cells [92]. Ning et al. demonstrated that circABCA1 promotes the proliferation and migration of clear cell renal cell carcinoma (ccRCC) both in vitro and in vivo through a mechanism dependent on SCARB1. Extending these findings, we further reveal that circABCA1 drives M2‐like macrophage polarisation and exerts a pro‐tumorigenic effect by enhancing SCARB1‐mediated cholesterol acquisition by ccRCC cells from the tumour microenvironment [57].

These findings underscore the significant regulatory roles of circRNAs in fatty acid transportation in cancer cells. However, the interactions between circRNAs and other lipid transportation‐associated genes, such as CD36, additional FABPs and LDLR, remain unclear and warrant further investigation for validation in future studies.

### 4.2 circRNAs Regulate Key Enzymes of Lipid Metabolism

It is not uncommon for tumour cells to increase lipid metabolism as a result of increased metabolic demands, regardless of whether exogenous lipid sources are available or there is a serum‐derived lipid deficiency within the tumour microenvironment [4]. Fatty acid and cholesterol metabolism involve numerous steps and several key enzymes participate in these processes [13, 19]. Recently, circRNAs have emerged as regulators of these key enzymes involved in lipid metabolism in cancer cells (Table 1 and Figure 3).

Acetyl‐CoA carboxylase alpha (ACACA), the rate‐limiting enzyme in fatty acid synthase, facilitates the carboxylation of CO2 and the conversion of acetyl‐CoA to malonyl‐CoA [93]. ACACA has been implicated in various cancers [94, 95, 96]. CircKIF18B_003 was overexpressed in prostate cancer tissues, and this overexpression correlated with unfavourable patient survival outcomes. Furthermore, it was functionally linked to dysregulated lipid metabolism. This oncogenic function is achieved by sponging miR‐370‐3p to elevate ACACA expression. Targeting this pathway, a combined treatment of ND‐630 and docetaxel significantly inhibited the growth of prostate cancer cells and xenografts [58].

ACCs serve as the pivotal rate‐limiting enzymes in fatty acid synthesis, catalysing the carboxylation of acetyl‐CoA to malonyl‐CoA in human cells [97]. Yang et al. demonstrated that circCAPRIN1 interacts with an interferon‐regulated transcription factor signal transducer and activator of transcription 2 (STAT2). This interaction results in the activation of ACC1 transcription, thereby regulating lipid metabolism, proliferation, migration and epithelial‐mesenchymal transition of CRC cells in vitro and in vivo [59]. Furthermore, circPCNXL2 was upregulated in papillary thyroid cancer (PTC), where its expression positively correlated with increased proliferation of PTC cells both in vitro and in vivo. Mechanistically, circPCNXL2 enhances ACC1 protein activity by reducing its phosphorylation at Ser79, thereby promoting the synthesis of fatty acids—including free fatty acids and triglycerides—to meet cellular energy demands and support tumour growth [60].

FASN, a crucial enzyme in the de novo synthesis of endogenous long‐chain fatty acids, catalyses the condensation of one molecule of acetyl‐CoA with 7 molecules of malonyl‐CoA to form 16‐carbon palmitate (C16:0) [98]. Elevated levels of FASN have been observed in numerous cancers including CRC, cervical cancer and breast cancer, and its expression correlates positively with cancer progression [65, 99, 100]. In pancreatic cancer samples, circ_0018909 has been found to be increased and acts by sequestering miR‐545‐3p, thereby increasing FASN expression. And pancreatic cancer cells are significantly more likely to proliferate, migrate, invade, undergo epithelial‐to‐mesenchymal transitions (EMT), and undergo apoptosis because of this mechanism in vitro and in vivo [61]. In oesophageal squamous cell carcinoma (ESCC), circHIPK3 acts as a ceRNA for miR‐637, thereby upregulating FASN expression and fatty acid synthesis—a mechanism which, when disrupted by circHIPK3 knockdown, significantly inhibits cancer cell proliferation, colony formation, migration and invasion in vitro, and tumour growth in vivo [62]. A study in intrahepatic cholangiocarcinoma (ICC) reported that circMBOAT2, which is frequently upregulated in ICC tissues and correlates with malignant features, promotes tumour progression. Its knockdown inhibited the growth and metastasis of ICC cells and xenografts. Mechanistically, circMBOAT2 stabilises PTBP1 by protecting it from ubiquitin/proteasome degradation. Consequently, PTBP1 regulates the export of FASN mRNA, reprogramming lipid metabolism and redox homeostasis in ICC cells [63]. In breast cancer, circWHSC1 was upregulated in tumour tissues compared to adjacent non‐tumour tissues, which correlated with higher tumour stage, lymphatic metastasis and poorer patient survival. Furthermore, functional studies demonstrated that circWHSC1 overexpression amplified the proliferation, migration and invasion of breast cancer cells and boosted xenograft tumour growth. This oncogenic function is mediated by its role as a ceRNA that sponges miR‐195‐5p, thereby upregulating FASN expression and activating the AMPK/mTOR pathway [64]. Additionally, circZFAND6 functions similarly as a ceRNA by sponging miR‐647 to enhance FASN expression, thereby promoting proliferation and metastasis of breast cancer cells in vitro and in vivo [65].

A member of the acyl‐coenzyme A synthetase (ACSL) family, long‐chain ACSL1 plays a crucial role in activating fatty acids ranging from 12 to 20 carbon atoms in length [101, 102]. Numerous studies related to ACSLs have indicated that ACSL1 is implicated in cancer progression and is associated with poor prognosis [103, 104]. Silencing of circPDHX in prostate cancer (Pca) cells has been shown to suppress cell fatty acid metabolism, proliferation and migration in vitro and PCa tumour growth in vivo. Mechanistically, miR‐497‐5p is sucked into circPDHX, resulting in increased ACSL1 expression [66]. Therefore, by promoting miR‐497‐5p/ACSL1 expression, circPDHX promotes prostate cancer cell proliferation [66].

An enzyme called fatty acid desaturase 2 (FADS2) plays a pivotal role in the biosynthesis of polyunsaturated fatty acids (LC‐PUFAs), catalysing the Δ6, Δ8 and Δ4 desaturation pathways [105, 106]. In osteosarcoma (OS), the expression of HSA_circ_0000073 is enhanced in tumour tissue and promotes lipid synthesis in OS cells by downregulating miR‐1184, increasing the levels of FADS2. Moreover, silencing of HSA_circ_0000073 inhibited lipid synthesis in vivo in a xenograft mouse model [67].

A monooxygenase involved in the cholesterol synthesis pathway, squalene epoxidase (SQLE) converts squalene to 2,3‐epoxy squalene [107, 108]. There is accumulating evidence indicating SQLE is frequently overexpressed in various cancers and correlates with poor patient prognosis in these malignancies [109, 110]. Qian et al. demonstrated that knockdown of circ_0000182 significantly inhibits cell proliferation and cholesterol synthesis in stomach adenocarcinoma (STAD) cells by sequestering miR‐579‐3p, thereby promoting SQLE expression [68].

Sterol O‐acyl‐transferase 1 (SOAT1) plays a critical role in maintaining cellular cholesterol homeostasis by catalysing the synthesis of cholesterol esters from cholesterol and long‐chain fatty acids within cells [111]. Our research team has previously identified elevated levels of circLDLR in CRC tissues, which correlated closely with the malignant progression and poor prognosis of CRC patients. Additionally, we demonstrated that by binding to miR‐30a‐3p, circLDLR enhances cell growth, metastasis and cholesterol metabolism in CRC cells in vitro and in vivo, thereby inhibiting miR‐30a‐3p's ability to downregulate SOAT1 expression [69].

Phospholipases (PLC, PLD and PLA) constitute a heterogeneous group of enzymes with the collective function of hydrolyzing phospholipids, crucial constituents of cell membranes [112]. CircNFIB was identified as a downregulated circRNA in breast cancer by Zhong et al. This circRNA modulates AA synthesis through regulation of phospholipases, which inhibits proliferation and invasion of breast cancer cell lines. Moreover, circNFIB inhibited tumour growth and metastasis in vivo [70].

Glycerophosphocholine phosphodiesterase 1 (GPCPD1) is a pivotal enzyme involved in choline and phospholipid metabolism [113], catalysing the hydrolysis of sn‐glycero‐3‐phosphocholine into glycero‐3‐phosphate and choline [114]. Huang et al. observed that circSNX6 expression was elevated in sunitinib‐resistant renal cell carcinoma (RCC) cells compared to their parental counterparts. In vitro and in vivo experiments demonstrated that circSNX6 functions as a molecular sponge, alleviating the suppressive effect of miR‐1184 on GPCPD1. In RCC cells, this interaction promotes the production of intracellular lysophosphatidic acid (LPA), increasing sunitinib resistance [71].

Carnitine palmitoyltransferase I A (CPT1A), belonging to the CPT1 family, serves as the crucial rate‐limiting enzyme in FAO, crucial for regulating cellular FAO and facilitating adaptation to environmental conditions [115]. CPT1A plays critical roles in cancer cell growth, survival and resistance to drugs [115, 116]. According to Wang et al., circ_0024107 derived from gastric cancer tissue‐derived mesenchymal stem cells (GC‐MSCs) induces FAO metabolic reprogramming and promotes gastric cancer lymphatic metastases in vitro and in vivo. They found that circ_0024107 upregulates CPT1A expression and FAO metabolic reprogramming in GC‐MSCs by acting as a sponge for miR‐5572 and miR‐6855‐5p [72]. Additionally, by activating mTORC1 signalling in CLL, circTET2 stabilises heterogeneous nuclear ribonucleoprotein C (HNRNPC), increasing lipid metabolism and proliferation of CLL cell lines [73].

Furthermore, circ‐NF1‐419 overexpression induced apoptosis in astroglioma U87 cells and influenced lipid metabolism through pathways involving glycerophospholipid metabolism and retrograde endocannabinoid signalling. Although the precise mechanisms by which circ‐NF1‐419 modulates lipid metabolism remain unclear, an observation was made that it regulates the expression of key metabolic enzymes including L‐type voltage‐operated calcium channels (L‐VOCC), phospholipase C‐β3 (PLCβ3), Mucin1, cationic amino acid transporter 4 (CAT4), cationic amino acid transporter 1 (CAT1) and A kinase (PRKA) anchor protein 4 (AKAP4) in U87‐NF1‐419 cells and U87 mouse tumour [74].

Overall, these discoveries highlight the pivotal role of circRNAs in regulating key enzymes involved in the metabolism of lipids, thereby influencing courses such as fatty acid and cholesterol metabolism, as well as impacting proliferation, metastasis and drug resistance in specific tumour cell types.

### 4.3 circRNAs Influence Regulators of Lipid Metabolism

Lipid metabolism is an intricate cellular process regulated by various factors including SREBPs, Liver X receptors alpha (LXRα) and beta (LXRβ) [117, 118]. To promote tumour progression, tumour cells can manipulate the expression or function of these regulators to influence lipid metabolism in the tumour microenvironment. This review emphasises the impact of circRNAs on the regulation of lipid metabolism regulators in cancer contexts (Table 1).

In cells, SREBPs regulate transcriptional activation of enzymes involved in fatty acid synthesis, cholesterol synthesis, triglyceride synthesis and phospholipid synthesis [117]. As master regulators of cholesterogenesis and lipogenesis, SREBPs are pivotal in cellular lipid metabolism regulation [117]. CircPRKAA1, originating from the AMP‐activated protein kinase (AMPK) α1 subunit, has been identified to enhance the stability of mature SREBP‐1 (mSREBP‐1) by using the Ku80/Ku70 heterodimer to promote the formation of a tetrameric complex [75]. Furthermore, the circPRKAA1 gene activates transcription of ACC1, ATP citrate lyase (ACLY), stearoyl‐Coenzyme A desaturase 1 (SCD1) and FASN genes by recruiting mSREBP‐1, which increases fatty acid synthesis and facilitates cancer growth in vitro and in vivo [75]. Wang et al. recently discovered that cytoplasmic circMyc interacts directly with the HuR protein, augmenting HuR binding to SREBP1 mRNA and increasing its stability in cells of triple‐negative breast cancer (TNBC). Additionally, a nuclear circMyc binds to the Myc protein at the SREBP1 promoter, resulting in enhanced transcription of SREBP1. Consequently, elevated SREBP1 levels promote the expression of downstream lipogenic enzymes, thereby promoting lipogenesis, cell proliferation and invasion in vitro and tumour growth in mice [76]. These studies underscore the critical role of circRNAs as regulators of SREBP‐1 expression and stability, influencing lipid metabolism in certain cancer contexts.

As a critical regulator of cholesterol metabolism, Insulin‐induced gene 1 (INSIG1) influences sterol synthesis through its roles in modulating SREBP activation and HMGCR degradation [119, 120, 121]. Recently, Xiong et al. identified a novel hypoxia‐responsive circular RNA, circINSIG1, which encodes a 121 amino acid protein known as circINSIG1‐121. This protein was found to enhance the K48‐linked ubiquitination of INSIG1 by recruiting the ubiquitin E3 ligase complex CUL5‐ASB6. This mechanism ultimately promotes cholesterol biosynthesis, thereby facilitating CRC cell proliferation and metastasis in vitro and in vivo [77].

Perilipin 3 (PLIN3), a member of the perilipin family, contributes to the formation and accumulation of lipid droplets [122]. Circ_0086414 was found to inhibit proliferation, metastasis and lipid accumulation in clear cell renal cell carcinoma (ccRCC) cells in vitro and in vivo by modulating the miR‐661/Erb‐b2 receptor tyrosine kinase 2 (ERBB2) axis, thereby suppressing the expression of the lipid metabolism regulator PLIN3 [78].

### 4.4 circRNAs Modulate Lipid Metabolism via Cancer‐Related Signalling Pathways

The AMPK pathway functions as a core regulator maintaining cellular energy homeostasis by coordinating various metabolic processes, such as the tricarboxylic acid (TCA) cycle, lipogenesis, glycolysis, cell cycle progression and mitochondrial dynamics [123, 124]. AMPK activation has been shown to regulate both anabolism and catabolism through direct phosphorylation of proteins and modulation of gene transcription in pathways involving lipid synthesis, oxidation and lipolysis [125]. AMPK activation is frequently observed in various cancers, suggesting its potential as a therapeutic target in cancer treatment [123]. The AMPK pathway in cancer is influenced by several circRNAs [126, 127, 128]. The purpose of this review is to examine the impact of the circRNA/AMPK axis on lipid metabolism in cancer (Table 1).

Li and colleagues reported that circACC1, derived from the human ACC1 gene, stabilised and enhanced the AMPK enzymatic activity in CRC cells, thereby regulating FAO and glycolysis, causing significant alterations in cellular lipid storage. Moreover, silencing circACC1 inhibited cancer cell proliferation and suppressed tumour growth in an HCT116 xenograft model [79]. Additionally, circLARP1B overexpression promoted fatty acid synthesis, cellular metastasis and lipid accumulation in HCC cells in vitro and in vivo through the heterogeneous nuclear ribonucleoprotein D (HNRNPD)–liver kinase B1 (LKB1)‐AMPK pathway [80]. These findings highlight the critical role of circRNA‐mediated AMPK pathway activation in lipid metabolism regulation and cancer progression.

Hedgehog signalling is critical to the patterning of embryonic tissues, to the regeneration of postembryonic tissues, and to the development of cancer cells [129]. Previous research has linked Hedgehog pathway defects to metabolic dysfunctions, including alterations in lipid metabolism [130, 131]. Consequently, modulation of the Hedgehog pathway can disrupt lipid metabolism balance in cancer cells, influencing cancer progression. The results of the study by Katsushima et al. suggest that circ_63706 increases total ceramide and oxidised lipid levels while reducing total triglyceride levels in children with sonic hedgehog (SHH) subtypes of medulloblastomas. Moreover, inhibition of circ_63706 impedes the proliferation, migration and invasion of SHH medulloblastoma cells in vitro and attenuates their tumorigenesis and growth in vivo [81].

Studies have indicated that additional cancer‐related signalling pathways, including NF‐κB, PI3K/AKT/mTOR and JAK/STAT pathways, play roles in modulating lipid metabolism in cancers [132]. However, current research on how circRNAs regulate lipid metabolism in cancer through these pathways is limited. In this context, more research is needed to clarify the precise mechanisms of circRNA‐mediated regulation.

### 4.5 circRNAs Affects Lipid Metabolism by Regulating Other Factors

Moreover, the influence of circRNAs on lipid metabolism in cancer may intersect with other regulatory factors (Table 1). High‐mobility group AT‐hook 2 (HMGA2) belongs to the HMGA family of non‐histone chromatin‐associated proteins [133] and is implicated in promoting tumorigenesis across various cancers [134, 135]. Wu et al. demonstrated that inhibition of the circARL8B/miR‐653‐5p axis suppressed cell viability, migration, invasion and fatty acid metabolism in breast cancer cells in vitro and in vivo by reducing HMGA2 expression [82]. However, the specific mechanisms by which HMGA2 regulates fatty acid metabolism in breast cancer remain to be fully elucidated.

Calnexin (CANX) proteins are calcium‐binding lectin‐like molecular chaperones crucial for the proper folding of newly synthesised glycoproteins [136]. In PCa, amplification of CircFAM126A promotes cholesterol synthesis and malignant progression. This oncogenic effect, demonstrated both in cell lines and a mouse xenograft model, is mediated through the miR‐505‐3p/CANX pathway [83]. However, this study did not explore the direct association between CANX and cholesterol synthesis in PCa cells. It is hypothesised that CANX may function as a molecular chaperone facilitating the proper folding of enzymes involved in cholesterol synthesis, thereby contributing to cholesterol synthesis in PCa cells.

## 5 Conclusion and Future Perspectives

Recent studies have increasingly highlighted the regulatory roles of miRNAs and lncRNAs in lipid metabolism across various cancers [137, 138, 139, 140]. However, there remains limited understanding of how dysregulated circRNAs contribute to lipid metabolism. This review aims to explore the roles and mechanisms of circRNAs in lipid metabolism in cancer. We synthesised current knowledge on circRNAs closely related to cholesterol and fatty acid metabolism, detailing their modulation of lipid metabolism in tumours through regulation of lipid transportation‐associated genes, key lipid metabolism enzymes, lipid metabolism regulators and cancer‐related signalling pathways. Importantly, numerous unidentified circRNAs likely participate in cancer lipid metabolism, necessitating further investigation into their interactions and mechanisms. Moreover, circRNA‐mediated regulation of fatty acid and cholesterol metabolism is the focus of most current research; however, it is conceivable that several circRNAs may also impact other lipid classes such as sphingolipids, triglycerides and phospholipids, which are pivotal for cancer progression.

The roles and molecular mechanisms of circRNAs in regulating lipid metabolism in cancer have been comprehensively elucidated. As a result of their covalently closed loop structure, lipid metabolism‐related circRNAs have inherent stability, highlighting their potential as prognostic and diagnostic biomarkers for reprogrammed lipid metabolism in various cancers [141]. Furthermore, integrating these circRNAs with lipid metabolism‐associated genes could enhance the accuracy of cancer diagnosis and prognostic evaluations. Nevertheless, the clinical implementation of circRNAs requires further advancements. Larger sample sizes, extended follow‐up periods and in vivo experiments are essential to unveil the expression patterns and functional roles of lipid metabolism‐related circRNAs in cancers, thereby advancing molecular diagnostics.

Targeting circRNAs to intervene in tumour lipid metabolic reprogramming has emerged as a highly promising novel therapeutic strategy. circRNAs are hailed as ‘next‐generation star targets for RNA interference,’ owing to their exceptional intracellular stability (> 24 h) conferred by a covalent closed‐loop structure, their tumour‐specific and highly differential expression profiles and their unique capability to function as molecular ‘scaffolds’ or ‘sponges’ that efficiently orchestrate key pathways in lipid synthesis, desaturation and oxidation [9, 142]. Complementing this, shRNA/siRNA technology has matured considerably. Short sequences can be designed to span their unique back‐splicing junction (BSJ), enabling precise circRNA silencing while minimising off‐target effects on parental linear mRNAs [143, 144]. Furthermore, chemical modifications such as 2′‐O‐methylation and phosphorothioate backbones, coupled with conjugates like cholesterol or GalNAc, significantly enhance the serum stability of these therapeutics and confer liver‐ or tumour‐targeting specificity [143, 144]. Silencing these key circRNAs directly perturbs fatty acid metabolic processes, thereby reversing pro‐tumorigenic lipid reprogramming. By systematically integrating these advantages and confronting the challenges, targeting the circRNA/lipid metabolism axis charts a promising strategic direction for developing a new generation of cancer metabolism therapies.

Despite considerable optimism fueled by recent advances in elucidating the roles of circRNAs in lipid metabolism, significant knowledge gaps and technical hurdles persist, impeding both mechanistic understanding and therapeutic exploitation. At the technical level, the accurate identification and functional annotation of circRNAs remain major challenges. Generated via back‐splicing, circRNAs share extensive sequence overlap with their linear mRNA isoforms, rendering them highly susceptible to misidentification in standard RNA sequencing pipelines that are optimised for linear transcripts. Moreover, tools for functional validation of circRNAs remain underdeveloped. Conventional gene‐silencing strategies (e.g., siRNA or shRNA) typically target exonic sequences shared between circular and linear transcripts, thereby failing to achieve isoform‐specific knockdown. In the realm of therapeutic translation, although circRNAs possess intrinsic resistance to exonuclease‐mediated decay—a property that theoretically endows them with enhanced stability—their development as RNA therapeutics is hampered by persistent challenges in production and delivery. In vitro transcription‐based synthesis frequently yields heterogeneous products contaminated with double‐stranded RNA impurities, which can trigger innate immune responses. More critically, efficient and safe in vivo delivery remains a formidable barrier: current delivery platforms, including lipid nanoparticles and viral vectors, lack the precision required for robust, tissue‐specific targeting of therapeutically relevant sites such as the brain or solid tumours.

In summary, circRNAs play significant roles in reshaping lipid metabolism in cancer by participating in the ncRNA regulatory network through mechanisms such as miRNA sequestration, RNA binding protein interaction and potential protein translation [142]. Elucidating these mechanisms not only deepens our understanding of the metabolic rewiring that underpins tumorigenesis but also uncovers novel targets and strategies for therapeutic intervention.

## Author Contributions

**Zhiwei Miao:** writing – original draft (equal); writing – review and editing (equal). **Jingjing Cao** and **Xiaoyu Wang:** funding acquisition (equal); methodology (equal). **Tongguo Shi** and **Chunyu Zhang:** supervision (equal); visualisation (equal); writing – review and editing (equal).

## Funding

This work was supported by the Gusu Health Talents Program (GSWS2024060), Jiangsu Province Traditional Chinese Medicine and Technology Deveopment Plan Project (MS2023103), Natural Science Foundation of Nanjing University of Chinese Medicine (XZR2023098), and Suzhou Science and Technology Development Plan Project (SKY2022016).

## Conflicts of Interest

The authors declare no conflicts of interest.

## Data Availability

The data that support the findings of this study are available from the corresponding author upon reasonable request.
